# Challenges in the Management of Recurrent CNS Solitary Fibrous Tumors: A Case Report

**DOI:** 10.7759/cureus.68661

**Published:** 2024-09-04

**Authors:** Gaurav Bector, Shubam Trehan, Prateek Jain, Mahyar Toofantabrizi, Mandeep Kaur

**Affiliations:** 1 Medicine and Surgery, Dayanand Medical College and Hospital, Ludhiana, IND; 2 Internal Medicine, Dayanand Medical College and Hospital, Ludhiana, IND; 3 Internal Medicine, All India Institute of Medical Sciences, Rishikesh, IND; 4 Internal Medicine, MedStar Union Memorial Hospital, Baltimore, USA; 5 Hospital Medicine, Healthcare Corporation of America (HCA) Florida Capital Hospital, Tallahassee, USA

**Keywords:** pazopanib, systemic therapy, radiotherapy, surgical resection, metastasis, recurrence, central nervous system, solitary fibrous tumor

## Abstract

Solitary fibrous tumors (SFTs) of the central nervous system (CNS) are rare mesenchymal neoplasms with diverse histological characteristics ranging from benign to malignant. Their higher chance for metastasis and recurrence poses significant diagnostic and therapeutic challenges. In this study, we present a 53-year-old female with a recurrent SFT of the cervical spine that was diagnosed initially 12 years ago. The patient underwent repeated surgical resections including laminectomy and gamma knife radiosurgery, as well as temozolomide, bevacizumab, and pazopanib therapy. Despite these interventions, she experienced continuous disease progression, with the cancer spreading to vital CNS locations. This study demonstrates the locally invasive nature of CNS SFTs and their complicated treatments involving surgical excision, radiotherapy, and systemic chemotherapy. This study highlights the need for new therapeutic approaches, as the existing methods fall short in meeting all the requirements and continue to lag in targeted therapy research for CNS SFTs. Consequently, it is important to develop individualized treatment strategies for patients affected by such difficult conditions.

## Introduction

Solitary fibrous tumors (SFTs) are uncommon neoplasms of mesenchymal origin that may arise in diverse anatomical sites including the central nervous system (CNS). They were initially referred to as hemangiopericytomas and their annual incidence is about one per million people, which makes them extremely rare [1,2]. SFTs have a wide range of clinical spectrum depending on location, especially within CNS which mostly affects individuals in their fifth and sixth decades of life. Clinically they can present as headaches, neurological deficits, or signs of increased intracranial pressure. Their potential for local recurrence and distant metastasis complicates their long-term management and prognosis [3,4].

Histologic examination reveals the presence of spindle cells arranged randomly. Immunohistochemical (IHC) studies are positive for CD34, Bcl-2, CD99, and vimentin while being negative for S100, desmin, and cytokeratin. The Ki-67 index is associated with tumor aggressiveness and recurrence propensity but it might vary between different cases [5]. Primary treatment involves surgical removal though this can be hindered by critical CNS sites involved. Residual disease can be treated and the risk of recurrence can be reduced through the application of radiotherapy as well as radiosurgery like gamma knife surgery after surgical resection [6]. Despite this, there are high chances of recurrence and metastasis [7]. Chemotherapies that include temozolomide or bevacizumab have had limited success in terms of controlling disease progression [8]. One example is tyrosine kinase inhibitors, such as pazopanib that has proven to be effective in treating soft tissue sarcomas like SFTs. However, its use is usually limited by undesirable impacts like hypertension necessitating dose adjustment [9,10].

## Case presentation

**Figure 1:**
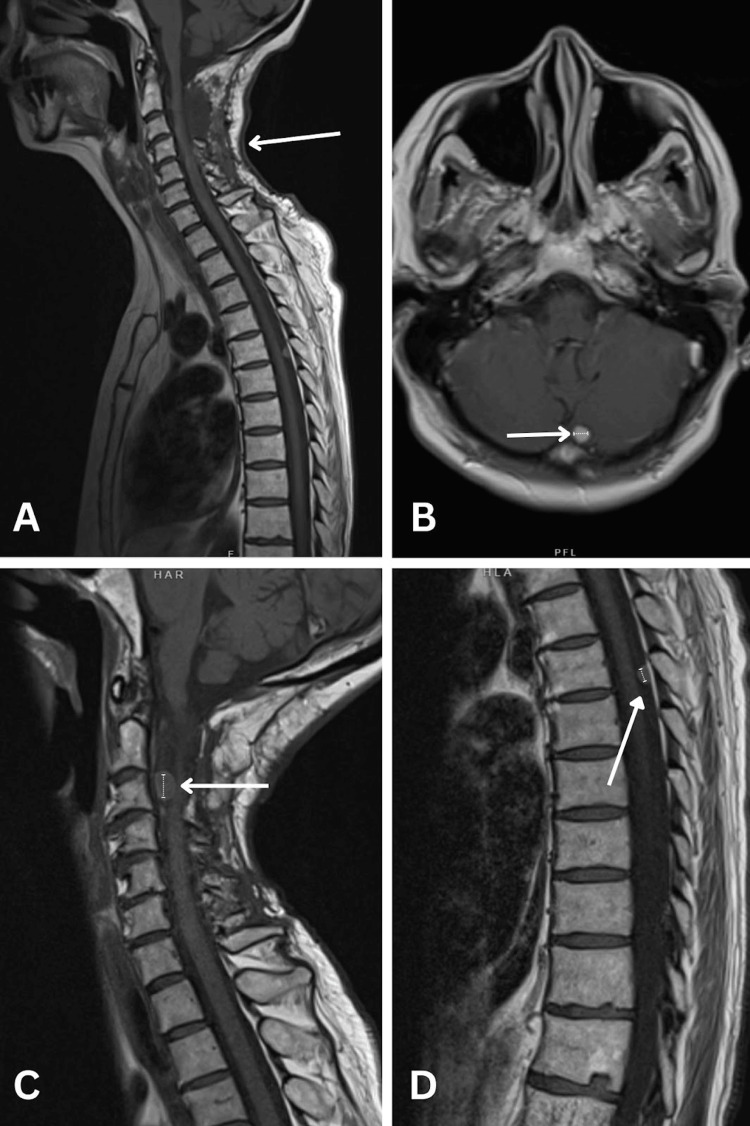
MRI images illustrating postoperative cervical spine changes and metastatic solitary fibrous tumor sites. (A) Sagittal T1 MRI sequence of spine without contrast showing postoperative tumor resection and laminectomy changes in the cervical spine. (B) Axial T1 MRI sequence of the brain without contrast showing 0.7 cm homogeneously enhancing left paramedian cerebellar mass (white arrow). (C) Sagittal T1 MRI sequence of cervical spine without contrast showing 1.2 cm ventral epidural mass at C3 with associated ventral thecal sac effacement (white arrow). No spinal canal or neural foraminal stenosis is appreciated. (D) Sagittal T1 MRI sequence of the thoracic spine without contrast showing 0.7 cm dorsal epidural mass at T5 without significant mass effect and cord deformity (white arrow). MRI: magnetic resonance imaging

A 53-year-old female from Puerto Rico with a significant history of recurrent SFT of the cervical spine, status post (S/P) multiple laminectomies, and currently on pazopanib 400 mg PO, once every 24 h, presented to the emergency department (ED) with complaints of fever, dyspnea, and fatigue due to community-acquired pneumonia. Her medical history included brain metastasis secondary to SFT of the cervical spine, treatment with chemoradiation, gamma knife surgery, as well as hypothyroidism for the past few years according to the patient. Twelve years ago, she was diagnosed with an extramedullary intradural SFT at the C1 level after presenting with a headache and neck pain, but limited information was available due to treatment outside. The tumor was resected successfully without radiation therapy. Histology revealed positivity for CD34, Bcl-2, CD99, and vimentin, with a Ki-67 index of up to 20%. Eight years later, the patient developed slowly progressive severe intermittent throbbing left-sided headaches. MRI showed metastasis to the cerebellum (without cerebellar signs), cervicomedullary junction, and multiple intracranial locations (Figures 1A-1D).

She underwent resection at the C1-C2 level, followed by radiation therapy. Subsequent surgeries included C2-C3 and C3-C4 laminectomies and gamma knife surgery, resulting in residual bilateral motor weakness (lower limbs > upper limbs).

The patient was started on temozolomide and bevacizumab, receiving 11 cycles of chemotherapy. During this period, a breast nodule was identified and resected, diagnosed as fibroadenoma. Despite chemotherapy, the tumor progressed, causing significant right-sided weakness and pain, necessitating a C5/C6 intradural extramedullary tumor resection with C5/C6 laminoplasty. Postsurgery, the temozolomide and bevacizumab regimen was discontinued, and pazopanib (400 mg PO, once every 24 h) was initiated. The dose was gradually increased to 800 mg but was later reduced to 400 mg due to a common side effect of hypertension. After nine months, follow-up scans showed increased tumor size at C3 and stable intracranial lesions. The patient experienced worsening bilateral upper extremity and right lower extremity weakness, prompting another C3 tumor resection with C2-C3 laminectomy. Postoperative imaging confirmed stable intracranial lesions and no remnant tumors. Her pneumonia was successfully resolved with a course of antibiotics. The timeline for the patient has been mentioned in the (Table 1).

**Table 1:** This table outlines the key events and treatments throughout the patient's clinical course. SFT: solitary fibrous tumor; C1, C2, C3, C4, C5, C6: cervical vertebrae 1 through 6; MRI: magnetic resonance imaging; LL: lower limb; UL: upper limb; PO: per oral; ED: emergency department; CD: cluster of differentiation; Bcl-2: B-cell lymphoma 2; Ki-67: Ki-67 proliferation index

Time point	Event
12 years ago	Diagnosis of extramedullary intradural SFT at C1 level. Initially presented with headache and neck pain.
	Tumor resection was performed without radiation therapy. Histology revealed positivity for CD34, Bcl-2, CD99, vimentin, Ki-67 index of up to 20%.
4 years ago	Development of severe intermittent headaches. MRI showed metastasis to the cerebellum, cervicomedullary junction, and multiple intracranial locations.
	Underwent resection at the C1-C2 level and radiation therapy.
3 years ago	Subsequent surgeries included C2-C3 and C3-C4 laminectomies and gamma knife surgery. Resulted in residual bilateral motor weakness (LL > UL).
	Started on temozolomide and bevacizumab, receiving 11 cycles.
2 years ago	Tumor progression continued, causing right-sided weakness and pain. Underwent C5/C6 intradural extramedullary tumor resection with C5/C6 laminoplasty.
	Temozolomide and bevacizumab regimen was discontinued. Pazopanib (400 mg PO daily) was initiated, later increased to 800 mg, and then reduced back to 400 mg due to hypertension.
9 months ago	Follow-up scans showed increased tumor size at C3 and stable intracranial lesions. Another C3 tumor resection with C2-C3 laminectomy was performed.
	Postoperative imaging confirmed stable intracranial lesions and no remnant tumors.
Present	Patient presented to the ED with fever, dyspnea, and fatigue due to community-acquired pneumonia. Pneumonia resolved with antibiotics.

## Discussion

This study highlights the aggressive and recurrent nature of solitary fibrous tumors (SFTs) of the central nervous system (CNS) and the subsequent failure of the present treatment modalities available.

Pathophysiology and diagnosis

Histologically, SFTs show a patternless arrangement of spindle cells, along with IHC staining positive for CD34, Bcl-2, CD99, and vimentin, and negative staining for S100, desmin, and cytokeratin. The Ki-67 index, for the initial tumor at the C1 level had a Ki-67 index of up to 20%, suggesting its aggressive growth and potential for recurrence (Table 2) [1-8].

**Table 2:** Pathological findings and significance. SFT: solitary fibrous tumor; CNS: central nervous system; CD34: cluster of differentiation 34; Bcl-2: B-cell lymphoma 2; CD99: cluster of differentiation 99; Ki-67: Ki-67 proliferation index

Pathological feature	Findings in patient	Significance
Histology	Spindle cells with patternless architecture	Typical of SFTs [1]
Immunohistochemistry		
CD34	Positive (focal)	Marker is often expressed in SFTs, indicating endothelial origin [2]
Bcl-2	Positive (focal)	Anti-apoptotic protein, often expressed in SFTs [2]
CD99	Positive (diffuse)	Marker associated with SFTs, though not specific [3]
Vimentin	Positive (diffuse)	Marker for mesenchymal origin, commonly positive in SFTs [3]
Pan-cytokeratin	Negative	Helps rule out the epithelial origin of the tumor [4]
S100	Negative	Helps rule out neural crest origin of the tumor [4]
CD56	Negative	Marker for neural origin, its negativity supports the diagnosis of SFT rather than neurogenic tumor [4]
Ki-67 proliferation Index	Up to 20%	Indicates high proliferative activity, correlates with aggressive behavior and potential for recurrence [5]
Transformation	Grade 3 sarcoma	Indicates malignant transformation with a higher potential for metastasis and poor prognosis [6]
Recurrence and metastasis sites	Multiple CNS locations (cerebellum, cervicomedullary junction, intracranial)	Reflects the aggressive nature and metastatic potential of high-grade SFTs [7]
Molecular Markers	Not specified in case	Molecular profiling could provide additional insights into tumor behavior and potential therapeutic targets [8]

When SFT involves the CNS, it commonly presents with symptoms of increased intracranial pressure, including persistent headaches and neurological deficits. The tumor's progression to a grade 3 sarcoma and its capacity for CNS metastasis highlight its aggressive and malignant behavior [4].

Treatment challenges and strategies

Complete surgical resection with clear margins is difficult due to the critical locations of these tumors. In our patient, multiple surgeries were required to manage recurrences and metastases, emphasizing the need for a multidisciplinary approach. Despite multiple surgeries, the patient continued to experience tumor progression and recurrence [5].

The use of gamma knife radiosurgery for critically located tumors further stresses the role of targeted radiotherapy in the management of SFTs [6]. Studies have shown that adjuvant radiotherapy can prevent local recurrence, while its impact on overall survival remains unclear [7]. Thanks to recent progress in molecular characterization, the identification of the NAB2-STAT6 fusion oncogene has emerged as a specific cytogenetic hallmark for SFT, involved in the overexpression of vascular endothelial growth factor (VEGF). These specific biological features encouraged the successful assessment of antiangiogenic drugs. Overall, antiangiogenic therapies showed a significant activity toward SFT in the advanced/metastatic setting. Nevertheless, these promising results warrant additional investigation to be validated, including randomized phase III trials and biological translational analysis, to understand and predict mechanisms of efficacy and resistance. While the therapeutic potential of immunotherapy remains elusive, the use of antiangiogenics as first-line treatment should be considered.

Systemic therapies

Systemic therapy regimens of temozolomide and bevacizumab frequently used for advanced or metastatic SFTs showed limited efficacy in our case. This correlates with results from other studies that report variable response to chemotherapy in SFTs [8]. Tyrosine kinase inhibitors, such as pazopanib, target angiogenesis, which is crucial for tumor growth and metastasis, by inhibiting vascular endothelial growth factor (VEGF) receptors. Our patient was maintained on pazopanib 400 mg after a few initial dose adjustments due to hypertension, following the discontinuation of temozolomide and bevacizumab. It is noteworthy that despite some control of the disease, the tumor progression continued, highlighting the limitations of current systemic therapies for SFTs [9]. Various studies have shown that pazopanib can stabilize disease progression in some patients but its efficacy is often limited by side effects (Table 3) [1,5,8-12].

**Table 3:** Molecular targets and treatment strategies for solitary fibrous tumors. EGFR: epidermal growth factor receptor; VEGF: vascular endothelial growth factor; PDGFR: platelet-derived growth factor receptor; KIT: proto-oncogene c-kit; mTOR: mammalian target of rapamycin; NAB2: neuroblastoma amplified sequence 2; STAT6: signal transducer and activator of transcription 6

Molecular target	Treatment agent	Mechanism of action	Clinical findings	Studies
EGFR	Erlotinib, gefitinib	Tyrosine kinase inhibitors targeting EGFR	Limited data on efficacy, potential target in cases with EGFR overexpression	Martin-Broto et al. (2021) [1]
Immune checkpoint inhibitors	Pembrolizumab, nivolumab	Monoclonal antibodies targeting PD-1/PD-L1 pathway	Preliminary studies suggest potential benefits. Further research needed to confirm the efficacy	Stacchiotti et al. (2012) [5]
VEGF receptor	Pazopanib	Tyrosine kinase inhibitor targeting VEGF receptors, inhibiting angiogenesis	It induces disease stabilization in some patients but is often limited by adverse effects such as hypertension	Lee et al. (2019) [8]
PDGFR and KIT	Imatinib	Tyrosine kinase inhibitor targeting PDGFR and KIT receptors	Some efficacy was reported in controlling tumor growth in SFTs with PDGFR and KIT mutations, variable response based on tumor genetics	Park et al. (2011) [9]
mTOR pathway	Sirolimus, everolimus	mTOR inhibitors reducing cell proliferation and survival	Experimental use, potential benefit in cases with mTOR pathway activation, though the evidence is preliminary	Katz et al. (2016) [10]
VEGF receptor	Bevacizumab	Monoclonal antibody targeting VEGF, inhibiting angiogenesis	Limited efficacy in controlling tumor progression when used with temozolomide; better results in combination with other agents	Park et al. (2011) [11]
NAB2–STAT6 fusion oncogene	Antiangiogenic drugs	Target VEGF overexpression	Significant activity toward SFT in the advanced/metastatic setting; promising results warrant further investigation, including randomized phase III trials and biological translational analysis to understand mechanisms of efficacy and resistance	de Bernardi et al. (2022) [12]

Long-term surveillance and prognosis

The importance of regular follow-up imaging to monitor disease progression is crucial. Even with aggressive multimodal treatment in the present case, the patient experienced recurrent and progressive disease over 12 years. The prognosis for patients with CNS SFTs depends on the extent of surgical resection, tumor grade, and response to adjunctive therapies. High-grade tumors, such as the grade 3 sarcoma, are associated with poor outcomes [11].

Future directions

A novel therapeutic approach targeting SFT pathogenesis should be explored due to the limited response to current treatment modalities. Immunotherapy has the potential to change the outcomes of such tumors with high recurrence rates. Immune checkpoint inhibitors, such as pembrolizumab and nivolumab, enhance the immune response against tumors by blocking PD-1/PD-L1 pathways, which enhance the body’s immune response against tumors, may be effective in treating SFTs [5]. A personalized approach based on genetic and molecular profiles is beneficial in managing SFT like the NAB2-STAT6 fusion oncogene. This method enhances treatment efficacy and reduces side effects by focusing on the tumor’s unique molecular drivers. Molecular profiling allows precise choice of treatment plans, prediction of remedy response, and adjustment of plans to fight resistance. By aligning treatment with the tumor’s genetic makeup, personalized remedy improves consequences and minimizes negative outcomes, moving past a generalized treatment technique [12].

## Conclusions

The present case demonstrates the aggressive behavior of the CNS SFTs and the difficult management of these tumors. Despite multiple surgeries, radiation therapy, and systemic treatments, our patient's tumor recurred and progressed. This example signifies the importance of a comprehensive multidisciplinary approach to treatment as well as long-term surveillance for recurrence or metastasis. The fact that presently available systemic therapies are not very effective suggests that there is an immediate need for further investigation into better treatment options. Recent advancements in molecular biology and genetics may pave the way for novel therapeutic approaches like immunotherapy in CNS SFT patients.
